# Development of artificial neural network model for predicting the rapid maxillary expansion technique in children with cleft lip and palate

**DOI:** 10.3389/fdmed.2025.1530372

**Published:** 2025-04-15

**Authors:** Mohamed Zahoor Ul Huqh, Johari Yap Abdullah, Adam Husein, Matheel AL-Rawas, Wan Muhamad Amir W. Ahmad, Nafij Bin Jamayet, Mohammad Khursheed Alam, Mohd Rosli Bin Yahya, Siddharthan Selvaraj, Abedelmalek Kalefh Tabnjh

**Affiliations:** ^1^ International Research Fellow, Faculty of Dentistry, SEGi University, Petaling Jaya, Selangor, Malaysia; ^2^ Craniofacial Imaging Laboratory, School of Dental Sciences, Health Campus, Universiti Sains Malaysia, Kubang Kerian, Kota Bharu, Malaysia; ^3^ Dental Research Unit, Center for Global Health Research, Saveetha Medical College and Hospital, Saveetha Institute of Medical and Technical Sciences, Saveetha University, Chennai, India; ^4^ Prosthodontic Unit, School of Dental Sciences, Health Campus, Universiti Sains Malaysia, Kubang Kerian, Kota Bharu, Malaysia; ^5^ College of Dental Medicine, Department of Preventive and Restorative Dentistry, University of Sharjah, Sharjah, United Arab Emirates; ^6^ Department of Biostatistics, School of Dental Sciences, Health Campus, Universiti Sains Malaysia, Kubang Kerian, Kota Bharu, Malaysia; ^7^ Division of Clinical Dentistry (Prosthodontics), School of Dentistry, International Medical University, Bukit Jalil, Kuala Lumpur, Malaysia; ^8^ Orthodontic Division, College of Dentistry, Jouf University, Sakaka, Saudi Arabia; ^9^ Oral & Maxillofacial Surgery Department, Hospital Raja Perempuan Zainab II, Kota Bharu, Malaysia; ^10^ Department of Public Health Dentistry, Saveetha Dental College and Hospitals, Saveetha Institute of Medical and Technical Sciences, Saveetha University, Chennai, India; ^11^ Department of Dental Research Cell, Dr.D.Y. Patil Dental College & Hospital, Pune, India; ^12^ Department of Cariology, Odontology School, Sahlgrenska Academy, Gothenburg University, Gothenburg, Sweden; ^13^ Department of Applied Dental Sciences, Faculty of Applied Medical Sciences, Jordan University of Science and Technology, Irbid, Jordan

**Keywords:** mid-palatal suture, logistic regression, rapid maxillary expansion, neural network, cleft lip and palate

## Abstract

**Aim:**

The study aimed to determine the mid-palatal suture (MPS) maturation stages and to develop a binary logistic regression model to predict the possibility of surgical or non-surgical rapid maxillary expansion (RME) in children with unilateral cleft lip and palate (UCLP).

**Methods:**

A retrospective case control study was conducted. A total of 100 subjects were included. Data was gathered from the databases of Hospital Universiti Sains Malaysia and Hospital Raja Perempuan Zainab II, respectively. Cone beam computed tomography scans of both cleft and non-cleft individuals were utilized to determine the MPS maturation stages. Romexis software version 3.8.2 was used to analyze the images.

**Results:**

The results of the binary logistic regression model were utilized to establish the relationship between the probability (P) of a specific event of interest (P(Y = 1)) and a linear combination of independent variables (Xs) using the logit link function. Potential factors such as age, gender, cleft, category of malocclusion, and MPS were chosen which could play a role in predicting the technique of RME in children with UCLP and non-UCLP. A subset of these variables was validated via multilayer feed forward neural network (MLFFNN).

**Conclusions:**

The effectiveness of the hybrid biometric model created in this work, which combines bootstrap and BLR with R-syntax was evaluated in terms of how accurately it predicted a binary response variable. A validation method based on an MLFFNN was used to evaluate the precision of the generated model. This leads to a good outcome.

## Introduction

Rapid maxillary expansion (RME) is a commonly employed technique for individuals with cleft lip and palate (CLP) (1–3). Maxillary expansion is essential due to restricted transverse development of the maxillary arch (3–5). Thus, RME is a routine procedure in CLP patients to correct the maxillary and mandibular width discrepancies (1, 6, 7). RME can be achieved successfully without the need for surgery in children in the pre-adolescent to adolescent age group due to the non-fusion of the mid-palatal suture (MPS). The resistance to expansion increases in adulthood due to the ossification of the circummaxillary and MPS (8).

The MPS is abnormally lateral to the midline in complete unilateral cleft lip and palate (UCLP), and the cleft side segment has no sutural relationship with the non-cleft side maxilla. Few studies were conducted to examine if it was feasible to expand the maxilla before surgery or after alveolar bone grafting (ABG) (9, 10). The findings of these studies relied on the fact that a diastema between maxillary central incisors causes splitting of maxillary processes in premaxillary region of the MPS resulting in clinically significant maxillary opening.

It has been proposed that an alveolar cleft encompasses the area corresponding to the tooth bud of the maxillary lateral incisor, inhibiting the formation of an intermaxillary suture in the premaxilla region. Thus, individuals with complete alveolar clefts may have an MPS in the premaxilla. Although there is no consensus on whether the premaxillary suture occurs in cleft patients, investigations confirming the absence of a completely distinct premaxillary suture have been recognized as the “incisive fissure” (11–13). A thin suture called the incisive suture is located in the anterior region of the premaxilla and embryologically originated from the primitive palate. However, in children with CLP, the palatal suture system is disrupted. Only a limited number of studies have discussed expansion in complete UCLP patients, and the presence or absence of MPS in cleft patients still remains controversial (13). The resistance to expansion increases in adulthood due to the ossification of the circummaxillary and MPS.

The prevalence rate of Cleft lip (CL) with or without Cleft palate (CP) in Malaysia was 1 in 1,000, with 1 in 3,000 children having CL (14, 15). CLP affects about one out of every 611 newborns in Malaysia (16). As per the previous study it was found that about 77.8% of the CLP instances in Malaysia were unilateral (17).

In routine clinical practice, chronological age is a typical predictor used to identify whether traditional non-surgical rapid maxillary expansion (NSRME) or surgically assisted rapid maxillary expansion (SARME) is more appropriate (8). However, there is no strong agreement between the authors in the literature on the age at which SARME should be performed (18).

In previous studies a deep learning models were developed to diagnose CLP before birth and also for precise diagnosis (19, 20). There is a lack of evidence-based literature on machine learning models in predicting the appropriate RME technique.

In our study a hybrid method was developed by combining binary logistic regression (BLR) model with bootstrap and multilayer feed forward neural network (MLFFNN) using R-syntax. The importance of statistical techniques has grown as a result of the demand for precise clinical results and its expanding necessity. Hybrid biometry techniques can manage unstructured and missing data while producing relevant results despite small sample sizes, making them a viable alternative to traditional diagnosis in children with CLP. It increases analytical skills and makes it easier to provide accurate information (21). Hence, the aim of this study was to determine the MPS maturation stages and to develop a logistic regression model to predict the possibility of NSRME or SARME.

## Materials and methods

### Study design and data collection

This was a retrospective case control study that included 50 patients with complete UCLP from Kelantan region, Malaysia and 50 individuals as controls who visited specialized orthodontic clinics at Hospital Universiti Sains Malaysia (HUSM) and Hospital Raja Perempuan Zainab II (HRPZ-II), Kota Bharu, Kelantan.

The cone beam computed tomography (CBCT) images of both cleft and non-cleft individuals were used to detemine the MPS maturation stages.

Subjects with the following criteria were included: (1) Non-syndromic complete UCLP children, (2) Patients in which cheiloplasty and palatoplasty have been performed, (3) Patients with class I, II and III malocclusions prior to orthodontic treatment, (4) Patients whose required data is completely available in the database.

Subjects with the following criteria were excluded from the study: (1) Subjects with bilateral CLP and partial clefts, (2) Any patients with associated syndromes or health issues due to cerebral palsy, anxiety disorders, epilepsy and musculoskeletal disorders, (3) Patients who already underwent any orthodontic treatment, (4) Patients who have been treated with secondary ABG, (5) Distorted, unclear CBCT images.

Purposive sampling was carried out. CLP patients' data logs, and CBCT images were acquired from the HRPZ-II hospital database. A convenience sampling was done for non-cleft individuals, and data were gathered from HUSM's specialized orthodontic clinic database. The records of the patients who visited the HUSM and HRPZ-II from July 2011–May 2021 have been selected.

### Data acquisition and measurement calibration

The CBCT images that were collected using a standardized protocol were chosen. The image analysis was carried out using Romexis software version 3.8.2. The categorization provided was used to determine the radiographic phases of the MPS as per the classification described by Angelieri et al. (22). The MPS was divided into five stages based on the presence of intermaxillary bony lines. Cross-sectional images of standardized CBCT in axial slice were utilized to evaluate the different stages of MPS development.

Stage A: A straight, dense MPS line with minimal or no interdigitation.

Stage B: A high-density suture line that is shaped irregularly and has scallops.

Stage C: Two closely spaced parallel scalloped high-density suture lines.

Stage D: Maturation has advanced from the posterior to the anterior in the palatine bone, and there will be fusion of MPS.

Stage E: The MPS has fused within the maxilla. The real suture is concealed in at least part of the maxilla.

An orthodontist with extensive knowledge and expertise performed the calibration and training procedures. A total of 10 CBCT slices from patients with UCLP aged 8 to 16 years of both genders were randomly selected. The observers were given a detailed explanation of the morphological characteristics of each MPS maturation stage in a high-resolution presentation of the image using Microsoft PowerPoint which included 10 CBCT axial and sagittal slices.

**Figure 1:**
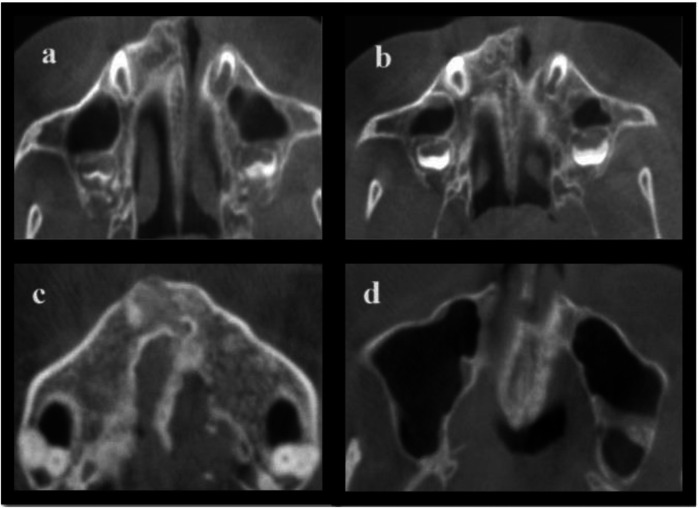
Represents the following stages of MPS in axial view of CBCT images: **(a)** A thick, visible, straight MPS line with minimal interdigitation; **(b)** A dense MPS line is seen that is irregular in shape; **(c)** Two scalloped, high-density MPS lines are seen; **(d)** The fusion of MPS is seen from posterior to anterior region of the palatine bone.

**Figure 2:**
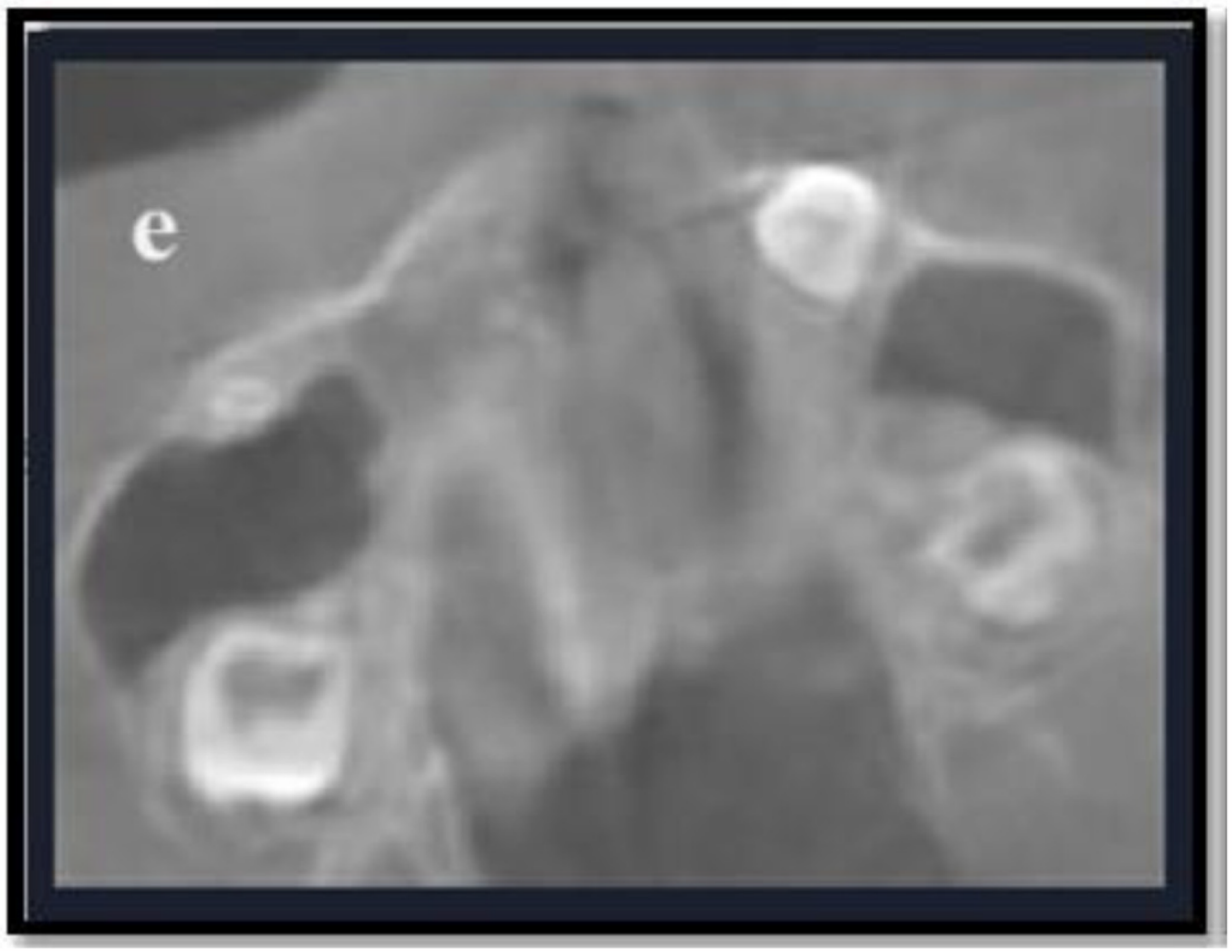
Represents the following stage of MPS in axial view of CBCT images: **(e)** Complete fusion of MPS is seen within the intermaxillary region.

Figures 1, 2 illustrate the different MPS maturation stages, respectively. A dataset was created for artificial intelligence (AI) modelling using R-syntax based on raw data obtained for MPS density measurements. The hybrid model developed utilizing R-syntax has been described below.

### Methodology building using R syntax – binary logistic regression (BLR)

**#/STEP 1**-Dataset for Biometry Modelling Study/#

Input =(“

Age Gender cleft category mps interc

15 0 1 3 4 1

14 1 1 3 5 1

16 1 1 3 4 1

15 1 1 3 3 0

15 0 1 3 4 1

15 0 1 3 4 1

……………

……………

14 1 1 3 5 1

14 0 1 3 4 1

15 1 1 3 3 0”)

data1 = read.table(textConnection(Input),header = TRUE)

#/Performing Bootstrap for 1000/#

mydata < - rbind.data.frame(data1, stringsAsFactors = FALSE)

iboot < - sample(1:nrow(mydata),size = 1000, replace = TRUE)

data < - mydata[iboot,]

#/Performing Multiple Logistics/#

#/Model Fitting/#

model = glm(interc ∼ Age + Gender + cleft + category + mps,

data = data,family = binomial(link=“logit”))

#/Performing Summary of the Model/#

summary(model)

exp(model$coefficients)

#/Overall *p*-value For Model/#

anova(model, update(model, ∼1), test = “Chisq”)

#/MultiLayer Perceptron Model/#

**#/STEP 2** - Install the Neuralnet Package/#

if(!require(neuralnet)){install.packages(“neuralnet”)}

library(“neuralnet”)

**#/STEP 3** - Checking For the Missing Values/#

apply(data, 2, function(x) sum(is.na(x)))

**#/STEP 4** - Max-Min Data Normalization/#

normalize < - function(x) {return ((x − min(x))/(max(x) − min(x)))}

maxmindf < - as.data.frame(lapply(data, normalize))

**#/STEP 5-**Determine the Training and Testing of the Dataset/#

#/70% for Training and 30% For Testing/#

index = sample(1:nrow(data),round(0.70*nrow(data)))

Training < - as.data.frame(data[index,])

Testing < - as.data.frame(data[-index,])

**#/STEP 6**-Plotting the Architecture of MLP Neural Network/#

nn < - neuralnet(interc∼Age + Gender + cleft + category + mps,data = Training,

hidden = c(3),act.fct = “logistic”,

linear.output = FALSE, stepmax = 1000000)

plot(nn) options(warn = −1)

nn$result.matrix

#/Testing The Accuracy of The Model- Predicted Result/#

**#/STEP 7**-Predicted Results Are Compared To The Actual Results/#

Temp_test < - subset(Testing, select = c(“Age”,“Gender”, “cleft”,“category”,“mps”))

head(Temp_test)

nn.results < - compute(nn, Temp_test)

**#/STEP 8-**Results

results < - data.frame(actual = Testing$interc,

prediction = nn.results$net.result)

**#/STEP 9**-Use The Predicted Mean Squared Error NN (MSE-forecasts the Network)

#/As a Measure of How Far the Predictions Are From The Real Data/#

predicted < - compute(nn,Testing[,1:5])

MSE.net < - sum((Testing$interc predicted$net.result)^2)/nrow(Testing)

**#/STEP 10-**Printing the Predicted Mean Square Error/#

MSE.net

###################Neural Network Parameter output#########################

**#/STEP 11**-Neural Network Parameter Output/#

library(neuralnet)

nn < - neuralnet(interc∼Age + Gender + cleft + category + mps,data = Training, hidden = 4,act.fct = “logistic”, linear.output = FALSE, stepmax = 1000000)

nn$result.matrix

######################Model Validation Calculation ########################

**#/STEP 12**- Model Validate/#

results < - data. frame(actual = Testing$interc,prediction = nn.results$net.result) results

summary(results)

#####################Model Accuracy Calculation ##########################

**#/STEP 13**- Model Accuracy/#

predicted1 = results$prediction*abs(diff(range(data$interc)))+min(data$interc)

#/Print (Predicted)/#

actual1 = results$actual*abs(diff(range(data$interc))) +min(data$interc)

#/Print(Actual1)/#

deviation = ((actual1-predicted1))

#/Print(deviation)/#

#/Mean Absolute Deviance/#

value = abs(mean(deviation))

print(value)

accuracy_in_percent=(1 − ((value)/100))*100

accuracy_in_percent

### Modelling of computational biometry with binary logistic regression

The construction of R syntax for the biometry hybrid approach consists of data bootstrapping, MLFFNN, and the binary logistic regression method, as well as the execution of the advanced strategy in three sections.

A statistical technique called binary logistic regression (BLR) examines the association between two binary response variables, such as the presence or absence of a disease in epidemiological studies or application of surgical or non- surgical method. It is typically used to investigate a current problem by assessing associated variables and projecting the likelihood that future cases may respond (23, 24).

Here the dependent variable used in logistic regression is a binary response variable, denoted as Y, which can take on values of 1 or 0. Examples of such variables include Yes or No (24).

**Figure 3:**
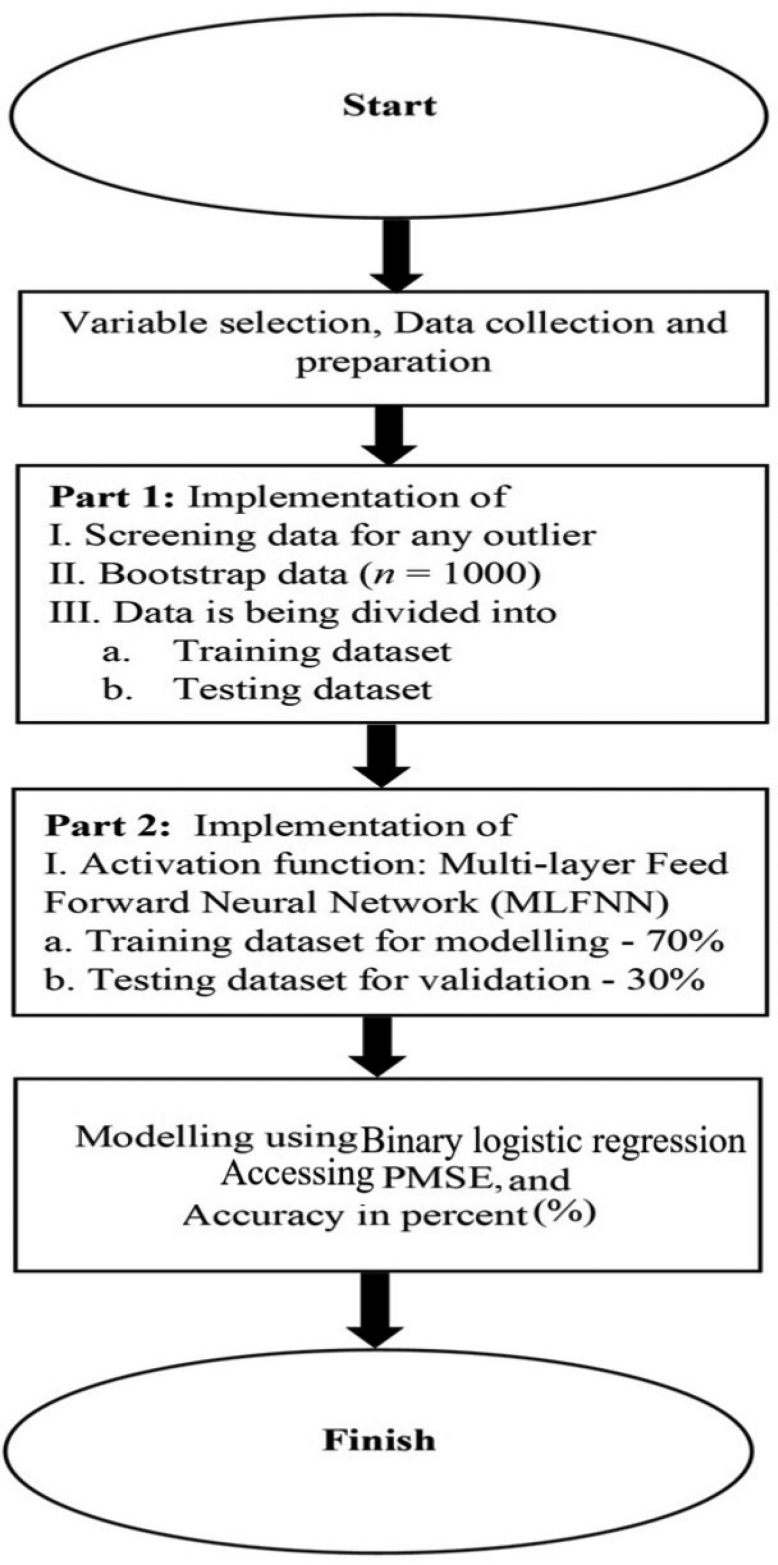
A flowchart of the suggested binary logistic regression modelling is provided to demonstrate the technique.

The logistic regression model to predict the possibility of SARME or NSRME via MPS morphology is presented here. The outcome of the variable is the binary response variable whether surgery or no surgery is required for RME and the explanatory variables are age (X1), gender (X2), cleft (X3), category of malocclusion (X4) and MPS stages (X5). The models are shown below based on RME treatment method. The flowchart for the proposed BLR model is displayed in Figure 3.

RMEij = 0 is non-surgical, if RMEij = 1 is surgical

The following model is defined as follows (25):

$$\overset{n}{\underset{i=1}{\sum }}Yi\;\;=\;\;\overset{n}{\underset{i=1}{\sum }}\pi (Xi)$$

$$\hat{\pi }(Xi)=\frac{e^{\beta 0+\beta 1(\mathrm{age})+\beta 2(\mathrm{gender})+\beta 3(\mathrm{cleft})+\beta 4(\mathrm{category})+\beta 5(\mathrm{mps})}}{1+e^{\beta 0+\beta 1(\mathrm{age})+\beta 2(\mathrm{gender})+\beta 3(\mathrm{cleft})+\beta 4(\mathrm{category})+\beta 5(\mathrm{mps})}}$$

### Statistical analysis

R-Studio software version 4.2.2 was used to analyze the collected data for associations linked to UCLP using the integrated defined syntax. Charts were utilized to present the analyzed data in addition to descriptive statistics like frequencies and means. Data analysis was done using a sophisticated technique, such as logistics regression with the MLFFNN which is a type of artificial neural network. The MLFFNN architecture consists of an input layer, hidden layer, and the output layer.

#### Bootstrap

Bootstrap first computes sample statistics from a random sample taken from the population. The bootstrap then draws a number of replacement samples after creating a pseudo-population by repeatedly copying the initial samples. The ability of the bootstrap to create a sample with the same size as the first sample, certain results repeated several times, and other results discarded. Samples produced by random sampling with substitution differ from the original sample. As the bootstrap draws the data with replacement, it produces statistics for each sample (26).

#### MLFFNN

**Figure 4:**
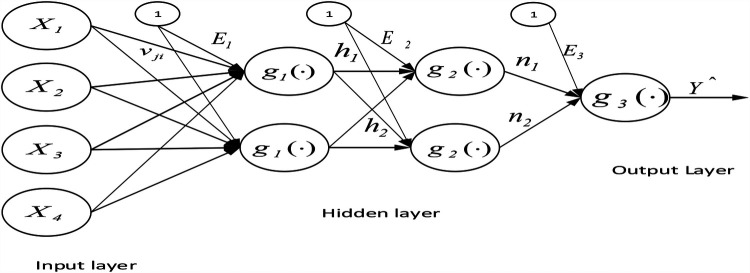
The general architecture of the MLFFNN with two hidden layers, N input nodes, H hidden nodes, and one output node.

The MLFFNN approach, a most popular artificial neural network design, was applied. MLFFNN is composed of the input, hidden, and output layers. Since there is just one dependent variable in the study sample, the output node of this analysis is singular. As seen in Figure 4, equation $\hat{Y}=g_{i}\left(\sum_{\;j=1}^{2}n_{j}+E_{3}\right)$ creates an MLFFNN with *N* input nodes, *H* hidden nodes, and a single output node.

The value $\hat{Y}$ is expressed as follows $\hat{Y}=g_{i}\left(\sum_{\;j=1}^{2}n_{j}+E_{3}\right)$, where g is an activation function and *E*_3_ is the bias for the output node.

Where *ν_ji_* is the output *weight* from input node *i* to hidden node *j,* and *j* = 1, 2 and *x_i_* are the independent variables. The variable chosen from the MLFFNN was used as input for the multiple logistic regression (27, 28).

## Results

The findings of binary logistic regression model were used to establish the relationship between the probability (P) of a certain event of interest [P(*Y* = 1)] and a linear combination of independent variables (*X*_s_), utilizing the logit link function. The logistic regression for binary response variable is defined in an equation (24):

Model:

Logit $\hat{g}(X_{i})=\beta_{0}+\beta_{1\mathrm{age}}+\beta_{2\mathrm{gender}}+\beta_{3\mathrm{cleft}}+\beta_{4\mathrm{category}}+\beta_{5\mathrm{mps}}$

$$P(Y=1|)\;=\frac{e^{g(Xi)}}{1+e^{g(Xi)}}\;$$

$$P(Y=1|)=\frac{e^{\beta_{0}+\beta_{1}(\mathrm{age})+\beta_{2}(\mathrm{gender})+\beta_{3}(\mathrm{cleft})+\beta_{4}(\mathrm{category})+\beta_{5}(\mathrm{mps})}}{1+e^{\beta_{0}+\beta_{1}(\mathrm{age})+\beta_{2}(\mathrm{gender})+\beta_{3}(\mathrm{cleft})+\beta_{4}(\mathrm{category})+\beta_{5}(\mathrm{mps})}}$$

“*Y*” is a binary response variable (*Y* = 1 or 0), e.g., Yes or No.

**Figure 5:**
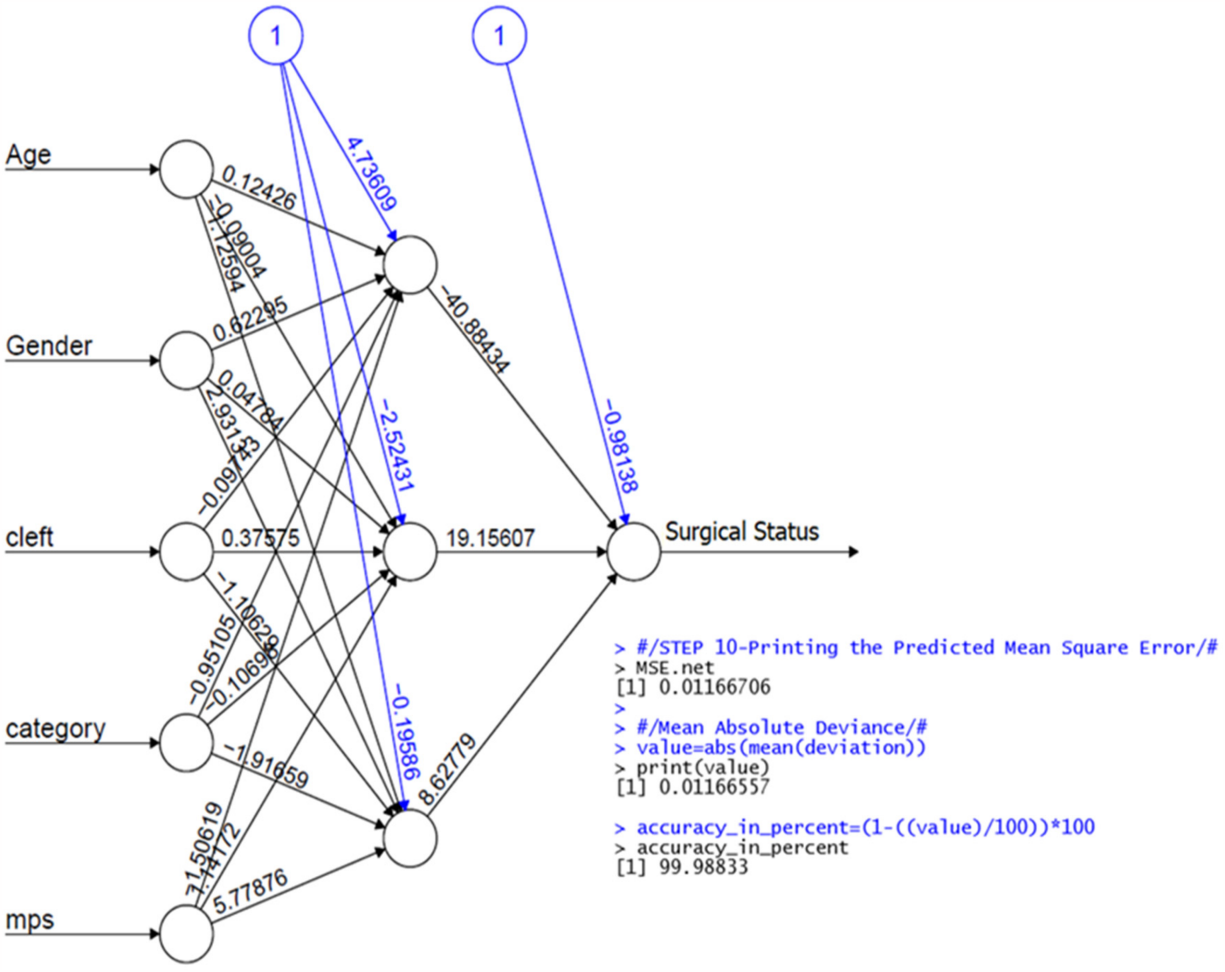
The architecture of the MLFFNN model with five input variables, one hidden layer and one output layer.

Potential variables such as age, gender, cleft, category of malocclusion, and MPS were chosen in the section which could play a role in predicting the technique of RME (whether SARME or NSRME) in children with UCLP and non-UCLP. Selected variables were validated by MLFNN in which age, gender, cleft, category of malocclusion, and MPS stages were used as input variables, and the binary response variable “*Y*” was used as the output variable as displayed in Figure 5.

A bootstrap method was employed by the hybrid approach to validate the factor. Table 1 provides a summary of the comprehensive findings. In this case, five factors were taken into account for the model's input based on their clinical importance. Age (−0.15268; *p* < 0.25), gender (0.48769; *p* < 0.25), cleft (1.17100; *p* < 0.25), category of malocclusion (−0.01592; *p* > 0.25) and MPS (6.08926; *p* < 0.25) are the variables.

**Table 1:** Results of binary logistic regression by combining the bootstrap method.

Variable	Estimate	Std. Error	Z-value	*p*-value
Age	−0.15268	0.03702	−4.124	3.73 × 10^−5^***
Gender	0.48769	0.12918	3.775	0.00016***
Cleft	1.17100	0.13874	8.440	<2 × 10^−16^***
Category of malocclusion	−0.01592	0.09721	−0.164	0.86991
MPS	6.08926	0.15784	38.580	<2 × 10^−16^***

Binary logistic Regression was applied.

*** Significant at the level of 0.25.

In Table 1, where the binary response variable “*Y*” is the dependent variable and the model's output. To predict the outcome, BLR was utilised. A Predicted Mean Square Error (PMSE) of 0.011% demonstrates the excellent performance of the proposed method. The analysis was successful if the PMSE value was low. The data used in this study was divided 70:30, meaning that 70% of the data was used for modelling and 30% for testing. The syntax for the proposed hybrid method in R is completely given under the subheading “The syntax in R for the proposed Hybrid Method” for BLR. The resulting model was created using R syntax. The variables, age, gender, cleft and MPS were statistically significant (*p* < 0.25) and have shown a strong correlation. However, the category of malocclusion was not statistically significant (*p* > 0.25).

The model assessment in this case was generated from the anticipated value. The forecast's accuracy may be assessed by comparing the expected and actual values. The test dataset was used to evaluate the model created from the training data set. The distance prediction method was applied to compare the real and predicted data. The model assessment method, which is available as R-syntax, allowed one to assess the effectiveness of the developed technique. The values between “actual” and “predicted” aren't significantly different. The “actual” and “predicted” values of the suggested model are shown in Table 2. The results showed that there was no statistically significant difference between “actual” and “predicted”. It implies that the most effective model is the proposed model. It has been demonstrated that equations may produce variables. Table 3 summarizes the sample distribution according to morphological development phases.

**Table 2:** Summary of “actual” and “predicted” value of the proposed binary model.

Actual	Predicted
0	0.535764
1	0.977713
0	0.476126
1	0.999858
1	0.999985

Indicator: 0 = non-cleft, 1 = cleft.

**Table 3:** Percentage distribution of study sample based on age, gender, and maturational stages.

Age(years) Maturation stages	8–10		11–13		14–16		Total
	M (%)	F (%)	M (%)	F (%)	M (%)	F (%)	
Stage A	2	1	4	1	0	0	8
Stage B	1	0	2	5	0	0	8
Stage C	3	2	2	1	7	5	20
Stage D	0	0	0	0	8	19	27
Stage E	0	0	0	0	14	23	37

## Discussion

The distinctive feature of individuals with CLP is the Midface deficiency. It was estimated that between 25 and 60 percent of children born with UCLP will require advancement of the maxilla to correct maxillary hypoplasia and to enhance the facial aesthetics (29). One of the most significant considerations in deciding how to treat a transverse maxillary constriction is determining whether the MPS is open or closed, which greatly influences the type of treatment that will be provided to the patient. This can be especially challenging in late-adolescent and early adult patients since there is no agreement in the literature on the minimum age required for effective palatal expansion (30–32). Despite the RME protocol's tremendous success in everyday clinical practise, there is no consensus guideline on the age restriction for MPS disjunction. This is mostly owing to the high potential of individuals of the same chronological age having varying maturational phases of the MPS (34). Numerous studies have demonstrated that chronologic age is not an important indicator in determining the difference in developmental stages of MPS fusion, especially in young adults (33, 35–38). Fishman found a limited association between chronological and skeletal age, highlighting the necessity for patient-specific indications of skeletal and facial maturation stages (39). The methodology described by Angieleri et al. (22) has been found to be a reliable method in assessing the MPS maturation stages.

The purpose of this research was to develop BLR model by incorporating advanced statistical tools such as bootstrap and MLFFNN using R-syntax based on input variables (age, gender, cleft, category of malocclusion and MPS stages) and RME technique as output variable.

The model's performance was then assessed using an additional dataset, and it performed excellently, with a Predicted Mean Square Error (PMSE) of 0.011%. The PMSE is a measure of the variance between our predictions and the actual outcomes. The PMSE value found in our investigation indicates a low probability of error.

The integration of hybrid biometry technology with logistic regression analysis played a crucial role in the advancement of this work. In these particular cases, a sophisticated statistical methodology that combines bootstrap and BLR employing R-syntax has demonstrated a high level of effectiveness in modelling, resulting in more precise outputs.

Furthermore, the model exhibited a high level of accuracy, precisely predicting the technique of RME in 99.98% of the instances.

Despite the fact that RME therapy is a commonly employed technique, only a few studies have examined treatment related alterations in children with CLP (40–43). The results of these studies have revealed differential anatomy in cleft individuals compared to non-cleft individuals may cause altered behavior of the maxilla in sagittal and vertical directions. From our investigation based on the maturational stages examined, the palatal fusion was more frequently observed in stages D and E suggesting the SARME for children aged between 14 and 16 years. The open sutures were noted in the remaining stages A, B and C respectively, indicating the NSRME for the children's age range 8–13 years.

Deep learning has made remarkable progress in medical imaging as artificial intelligence has advanced. In medical image classification, the convolutional neural network (CNN) has demonstrated great accuracy in numerous prior CBCT image classification tasks and is capable of extracting local features (44–46). The CNN's ability to recognize patterns and capture global information is limited by the local characteristics of the convolutional layer. Although the theoretical receptive field of deep pixels can cover the entire image, the actual receptive field is much smaller and also raises CNN's computational cost (47).

The advantage of this hybrid technique, when combined with the R syntax algorithm, have produced excellent research and the best outcomes with low computational cost particularly for the decision-maker.

For the clinical implementation of the model, instructional materials, and appropriate training must be provided to the clinical staff members. The product label should be created which will aid clinicians to understand when and how to properly utilize the model outputs in their clinical decisions.

## Conclusion

The hybrid biometric model developed in this study that include bootstrap and BLR utilizing R-syntax was used to test the model's efficacy in determining the prediction accuracy of a binary response variable. The accuracy of the resulting model was assessed using a validation technique that utilized a MLFFNN. This leads to a good outcome. In female children the highest percentage of MPS maturation stages D (27%) and E (37%) was found. The greater number of ossification was seen in stage D and E, respectively.

## Limitations of the study

The current study cannot identify whether similar effects were seen in individuals with different kinds of clefts. The data utilized in this study were gathered retrospectively from secondary sources. Furthermore, our study sample consisted exclusively of Malay and Chinese children of Malaysian descent. There were no participants of Indian origin. It is crucial to note that the study's findings and conclusions might not apply to other racial or ethnic groups. Further longitudinal studies incorporating several cleft care centers involving other ethnicities are required with a multicentre effort that could indicate a generalization of UCLP status in Malaysia when compared to children with other ethnic background. Additionally, collaboration with clinical facilities is needed to test and develop the model in real-world scenarios.

## Data Availability

The original contributions presented in the study are included in the article/Supplementary Material, further inquiries can be directed to the corresponding author/s.

## References

[1] Pan X Qian Y Yu J Wang D Tang Y Shen G. Biomechanical effects of rapid palatal expansion on the craniofacial skeleton with cleft palate: a three-dimensional finite element analysis. Cleft Palate Craniofac J. (2007) 44(2):149–54. 10.1597/05-161.1 17328641

[2] Lypka M Yen S Urata M Hammoudeh J. Solving convergent vector problems with internal maxillary distractors through the use of a fixed rapid palatal expander. J Oral Maxillofac Surg. (2012) 70(7):e428–30. 10.1016/j.joms.2012.03.013 22698298

[3] Yang CJ Pan XG Qian YF Wang GM. Impact of rapid maxillary expansion in unilateral cleft lip and palate patients after secondary alveolar bone grafting: review and case report. Oral Surg Oral Med Oral Pathol Oral Radiol. (2012) 114(1):e25–30. 10.1016/j.tripleo.2011.08.030 22732853

[4] Thompson JR. The cleft lip and palate problem. Angle Orthod. (1952) 22(3):137–41.

[5] Ferguson MWJ. Development of the face and palate. Cleft Palate Craniofacial J. (1995) 32(6):522–3. 10.1597/1545-1569_1995_032_0522_dotfap_2.3.co_2

[6] Holberg C Holberg N Schwenzer K Wichelhaus A Rudzki-Janson I. Biomechanical analysis of maxillary expansion in CLP patients. Angle Orthod. (2007) 77(2):280. 10.2319/0003-3219(2007)077[0280:BAOMEI]2.0.CO;2 17319763

[7] Gautam P Zhao L Patel P. Biomechanical response of the maxillofacial skeleton to transpalatal orthopedic force in a unilateral palatal cleft. Angle Orthod. (2011) 81(3):503–9. 10.2319/070110-367.1 21299384 PMC8923561

[8] Celebi F Akbulut S. Relationship between the position of maxilla and rapid maxillary expansion failure. South Eur. J. Orthod. Dentofac. Res.. (2020) 7(2):49–54. 10.5937/sejodr7-28432

[9] Ade O C de Albuquerque MD Filho LC. Rapid maxillary expansion after secondary alveolar bone graft in a patient with bilateral cleft lip and palate. Cleft Palate Craniofac J. (2004) 41(3):332–9. 10.1597/02-099.1 15151452

[10] da Silva Filho OG Boiani E de Oliveira Cavassan A Santamaria M Jr. Rapid maxillary expansion after secondary alveolar bone grafting in patients with alveolar cleft. Cleft Palate Craniofac J. (2009) 46(3):331–8. 10.1597/07-205.1 19642749

[11] Wood NK Wragg LE Stuteville OH Oglesby RJ. Osteogenesis of the human upper jaw: proof of the non-existence of a separate premaxillary centre. Arch Oral Biol. (1969) 14(11):1331–9. 10.1016/0003-9969(69)90206-4 4187720

[12] Behrents RG Harris EF. The premaxillary-maxillary suture and orthodontic mechanotherapy. Am J Orthod Dentofacial Orthop. (1991) 99(1):1–6. 10.1016/s0889-5406(05)81673-7 1986516

[13] Zahra S Samih H. Absence or presence of mid-palatal suture in patients with complete unilateral cleft lip and palate, (A retrospective study). Egypt Dent J. (2017) 63:1155–64. 10.21608/edj.2017.73887

[14] (IPDTOC) working group. Prevalence at birth of cleft lip with or without cleft palate: data from the international perinatal database of typical oral clefts (IPDTOC). Cleft Palate Craniofac J. (2011) 48(1):66–81. 10.1597/09-217 20507242

[15] Gopinath VK Samsudin AR Mohd Noor SNF Mohamed Sharab HY. Facial profile and maxillary arch dimensions in unilateral cleft lip and palate children in the mixed dentition stage. Eur J Dent. (2017) 11(1):76–82. 10.4103/ejd.ejd_238_16 28435370 PMC5379840

[16] Lau KL Ong SC Wan Sulaiman WS. Comparison between parents’ and patients’ satisfaction level towards cleft management using cleft evaluation profile. IIUM J Orofac Health Sci. (2021) 2(1):37–45. 10.31436/ijohs.v2i1.63

[17] Ali S Shah S Rahman Z Sahito M. Demographic data on the characterization of oral clefts in Malaysia. Pakistan Oral Dental J. (2015) 35:108–10.

[18] Angelieri F Franchi L Cevidanes LH Bueno-Silva B McNamara JA Jr. Prediction of rapid maxillary expansion by assessing the maturation of the midpalatal suture on cone beam CT. Dental Press J Orthod. (2016) 21(6):115–25. 10.1590/2177-6709.21.6.115-125.sar 28125147 PMC5278941

[19] Almoammar KA. Harnessing the power of artificial intelligence in cleft lip and palate: an in-depth analysis from diagnosis to treatment, a comprehensive review. Children (Basel). (2024) 11(2):140. 10.3390/children11020140 38397252 PMC10886996

[20] Baeza-Pagador A Tejero-Martínez A Salom-Alonso L Camañes-Gonzalvo S García-Sanz V Paredes-Gallardo V. Diagnostic methods for the prenatal detection of cleft lip and palate: a systematic review. J Clin Med. (2024) 13(7):2090. 10.3390/jcm13072090 38610855 PMC11012824

[21] Shafi N Bukhari F Iqbal W Almustafa KM Asif M Nawaz Z. Cleft prediction before birth using deep neural network. Health Informatics J. (2020) 26(4):2568–85. 10.1177/1460458220911789 32283987

[22] Angelieri F Cevidanes LH Franchi L Gonçalves JR Benavides E McNamara JA Jr. Midpalatal suture maturation: classification method for individual assessment before rapid maxillary expansion. Am J Orthod Dentofacial Orthop. (2013) 144(5):759–69. 10.1016/j.ajodo.2013.04.022 24182592 PMC4185298

[23] Cox DR Snell EJ. Analysis of Binary Data. 2nd ed. Oxford, UK: Routledge (2018).

[24] Srimaneekarn N Hayter A Liu W Tantipoj C. Binary response analysis using logistic regression in dentistry. Int J Dent. (2022) 2022:5358602. 10.1155/2022/5358602 35310463 PMC8924599

[25] Muna F Mohamad Ghazali FM Ahmad WMA Ahmad W Arif M Awang Nawi M Validate the factor from multiple logistic regression using artificial neural networks (ANNS) model: A case study of an elderly health status at receiving home care. Sapporo Med J. (2020) 54:1–11.

[26] Effron B Tibshirani RJ. An introduction to the Bootstrap. New York: Chapman & Hall (1993).

[27] Mohamed N Muhamad W Ahmad WMA Aleng N Ahmad M Matematik J Assessing the efficiency of multilayer feed-forward neural network model: application to body mass Index data. World Appl Sci J (2011):15(5):677–82.

[28] Aleng N Mohamed N Ahmad WMA Naing NN. A new strategy to analyze medical data using combination of M-estimator and multilayer feed-forward neural network model. Eur J Sci Res. (2012) 73:79–85.

[29] Elabbassy EH Sabet NE Hassan IT Elghoul DH Elkassaby MA. Bone-anchored maxillary protraction in patients with unilateral cleft lip and palate. Angle Orthod. (2020) 90(4):539–47. 10.2319/091919-598.1 33378498 PMC8028472

[30] McNamara JA Jr. Long-term adaptations to changes in the transverse dimension in children and adolescents: an overview. Am J Orthod Dentofacial Orthop. (2006) 129(4 Suppl):S71–4. 10.1016/j.ajodo.2005.09.020 16644422

[31] Liu S Xu T Zou W. Effects of rapid maxillary expansion on the midpalatal suture: a systematic review. Eur J Orthod. (2015) 37(6):651–5. 10.1093/ejo/cju100 25700989

[32] Jimenez-Valdivia LM Malpartida-Carrillo V Rodríguez-Cárdenas YA Dias-Da Silveira HL Arriola-Guillén LE. Midpalatal suture maturation stage assessment in adolescents and young adults using cone-beam computed tomography. Prog Orthod. (2019) 20(1):38. 10.1186/s40510-019-0291-z 31591660 PMC6779683

[33] Shayani A Merino-Gerlach MA Garay-Carrasco IA Navarro-Cáceres PE Sandoval-Vidal HP. Midpalatal suture maturation stage in 10- to 25-year-olds using cone-beam computed tomography-A cross-sectional study. Diagnostics (Basel). (2023) 13(8):1449. 10.3390/diagnostics13081449 37189552 PMC10137454

[34] Silva-Montero JC Faus-Matoses I Ribas-Pérez D Pourhamid H Solano-Mendoza B. Analysis of the frequency and correlated factors of midpalatal suture maturation stages in young adults, based on cone beam computed tomography imaging. J Clin Med. (2022) 11(23):6959. 10.3390/jcm11236959 36498534 PMC9740603

[35] Persson M Thilander B. Palatal suture closure in man from 15 to 35 years of age. Am J Orthod. (1977) 72(1):42–52. 10.1016/0002-9416(77)90123-3 267435

[36] Wehrbein H Yildizhan F. The mid-palatal suture in young adults. A radiological-histological investigation. Eur J Orthod. (2001) 23(2):105–14. 10.1093/ejo/23.2.105 11398548

[37] Knaup B Yildizhan F Wehrbein H. Age-related changes in the midpalatal suture. A histomorphometric study. J Orofac Orthop. (2004) 65(6):467–74. 10.1007/s00056-004-0415-y 15570405

[38] Korbmacher H Schilling A Püschel K Amling M Kahl-Nieke B. Age-dependent three-dimensional microcomputed tomography analysis of the human midpalatal suture. J Orofac Orthop. (2007) 68(5):364–76. 10.1007/s00056-007-0729-7 17882364

[39] Fishman LS. Chronological versus skeletal age, an evaluation of craniofacial growth. Angle Orthod. (1979) 49(3):181–9. 10.1043/0003-3219(1979)049<0181:Cvsaae>2.0.Co;2 225970

[40] Subtelny JD Brodie AG. An analysis of orthodontic expansion in unilateral cleft lip and cleft palate patients. Am J Orthod. (1954) 40:686–97. 10.1016/0002-9416(54)90057-3

[41] Isaacson RJ Murphy TD. Some efifects of rapid maxillary expansion in cleft lip and palate patients. Angle Orthod. (1964) 34(3):143–54.

[42] Tindlund RS Rygh P Bøe OE. Intercanine widening and sagittal effect of maxillary transverse expansion in patients with cleft lip and palate during the deciduous and mixed dentitions. Cleft Palate Craniofac J. (1993) 30(2):195–207. 10.1597/1545-1569_1993_030_0195_iwaseo_2.3.co_2 8452842

[43] Figueiredo DS Bartolomeo FU Romualdo CR Palomo JM Horta MC Andrade I Jr. Dentoskeletal effects of 3 maxillary expanders in patients with clefts: a cone-beam computed tomography study. Am J Orthod Dentofacial Orthop. (2014) 146(1):73–81. 10.1016/j.ajodo.2014.04.013 24975001

[44] Wang SF Xie XJ Zhang L Chang S Zuo FF Wang YJ Research on multi-class orthodontic image recognition system based on deep learning network model. Chin J Stomatology. (2023) 58(6):561–8. 10.3760/cma.j.cn112144-20230305-00070 37272001

[45] Hung KF Ai QYH Wong LM Yeung AWK Li DTS Leung YY. Current applications of deep learning and radiomics on CT and CBCT for maxillofacial diseases. Diagnostics. (2022) 13(1):110. 10.3390/diagnostics13010110 36611402 PMC9818323

[46] Duman ŞB Syed AZ Celik Ozen D Bayrakdar İ Salehi HS Abdelkarim A Convolutional neural network performance for sella Turcica segmentation and classification using CBCT images. Diagnostics. (2022) 12(9):2244. 10.3390/diagnostics12092244 36140645 PMC9498199

[47] Liu Y Yu J Han Y. Understanding the effective receptive field in semantic image segmentation. Multimedia Tools Appl. (2018) 77:22159–71. 10.1007/s11042-018-5704-3

